# *Streptomyces* strains modulate dynamics of soil bacterial communities and their efficacy in disease suppression caused by *Phytophthora capsici*

**DOI:** 10.1038/s41598-021-88495-y

**Published:** 2021-04-29

**Authors:** Sakineh Abbasi, Ayme Spor, Akram Sadeghi, Naser Safaie

**Affiliations:** 1 Department of Plant Pathology, Faculty of Agriculture, Tarbiat Modares University, Tehran, Iran; 2 Department of Agroecology, AgroSup Dijon, INRA, University de Bourgogne, University de Bourgogne Franche-Comte, Dijon, France; 3 Department of Microbial Biotechnology, Agricultural Biotechnology Research Institute of Iran (ABRII), Agricultural Research, Education and Extension Organization (AREEO), Karaj, Iran

**Keywords:** Plant sciences, Plant stress responses

## Abstract

The responses of rhizosphere bacterial communities of *Streptomyces* (SS14 and IT20 stains) treated-pepper plants following inoculation by *Phytophthora capsici* (PC) was investigated using Illumina MiSeq sequencing. Distinct modulation of the bacteriome composition was found for PC samples with the highest relative abundance (RA) of *Chitinophaga* (22 ± 0.03%). The RA of several bacterial operational taxonomic units (OTUs) was affected and caused changes in alpha and beta-diversity measures. In IT20, the RA of Cyanobacteria was enriched compared to SS14 (72%) and control samples (47%). Phylotypes belonging to *Devosia, Promicromonospora, Kribbella, Microbacterium, Amylocolatopsis,* and *Pseudomonas* genera in the rhizosphere were positively responding against the pathogen. Our findings show that the phosphate solubilizing strain IT20 has higher microbial community responders than the melanin-producing strain SS14. Also, positive interactions were identified by comparing bacterial community profiles between treatments that might allow designing synthetic bio-inoculants to solve agronomic problems in an eco-friendly way.

## Introduction

*Streptomyces* species, Gram-positive filamentous bacteria, are the most abundant and possibly the most important Actinomycetes^1^. Plant growth-promoting (PGP) *Streptomyces* strains colonize the rhizosphere/plant root and they could have potential as a bio-inoculant against biotic and abiotic stress conditions through different mechanisms^2,3^. In a previous study, PGP and biocontrol activity of *S. rochei* strain Y28 against *Fusarium oxysporum* f. sp. *lycopersici* race 3 causal agent of tomato *Fusarium* wilt was reported^2^. Antagonistic activity of *S. vinaceusdrappus* was previously reported against *Rhizoctonia solani* on tomato^4^ and *Pyricularia oryzae* on rice^5^. The soil-borne oomycete pathogen, *Phytophthora capsici* Leonian, causes the disease of pepper and several important crops^6,7^. Some strategies such as chemical treatment (Ridomil) and biological control have been endorsed to disease management^8,9^.

The disease suppression induced by biocontrol agents is related to interactions between the plant, pathogens, biocontrol agents, the surrounding microbial community, and the environment^10^. van Elsas et al.^11^ showed that an increase in soil bacterial diversity can reduce the relative abundance of pathogens and could be an efficient tactic in controlling plant diseases. Chen et al.^12^ revealed that microbial communities in the rhizosphere negatively correlated to the level of disease severity. Previous studies using biocontrol agents have mainly revealed that the dynamics of soil bacterial populations played a critical role in disease suppression caused by soil-borne fungal^13,14^.

The studies on *Phytophthora* blight disease suppression affecting pepper were conducted on the characterization and identification of native plant growth-promoting soil bacterial genera such as *Bacillus* and *Pseudomonas* and their antagonistic potential. The volatile organic compounds produced by bacterial antagonists exposed anti-oomycete effects^15,16^. Also, Li et al.^17^ reported that long-term application of organic fertilizers caused intense changes in soil microbial consortium possibly *Bacillus* antagonists and significantly suppressed pepper blight disease caused by *P. capsici*. However, very few investigations have been conducted to address the complex interactions among biocontrol strains of *Streptomyces* introduced, different genera of beneficial microbes, and native microbial communities in the rhizosphere of healthy plants. On the other hand, the effects of phosphate (P) solubility on microbe-microbe interactions and disease suppression are poorly understood. In the current study, we investigated the hypothesis that P solubilizing or melanin producing strain might boost the soil biodiversity, lead to changes in the abundance of indigenous microbial communities of the rhizosphere and distinctly suppress disease caused by *P. capsici*.

The aims of this study were to (1) screen in vitro antifungal activity of some *Streptomyces* species against *P. capsici* from microbial culture collection (2) evaluate *Phytophthora* blight disease suppression in pepper plants treated by two superior anti-oomycete isolates under sterile and non-sterile soil conditions (3) the rhizosphere community analysis using culture-dependent and NGS sequencing.

## Results

### Growth inhibition toward *P. capsici* and characterization of *Streptomyces* isolates

**Figure 1:**
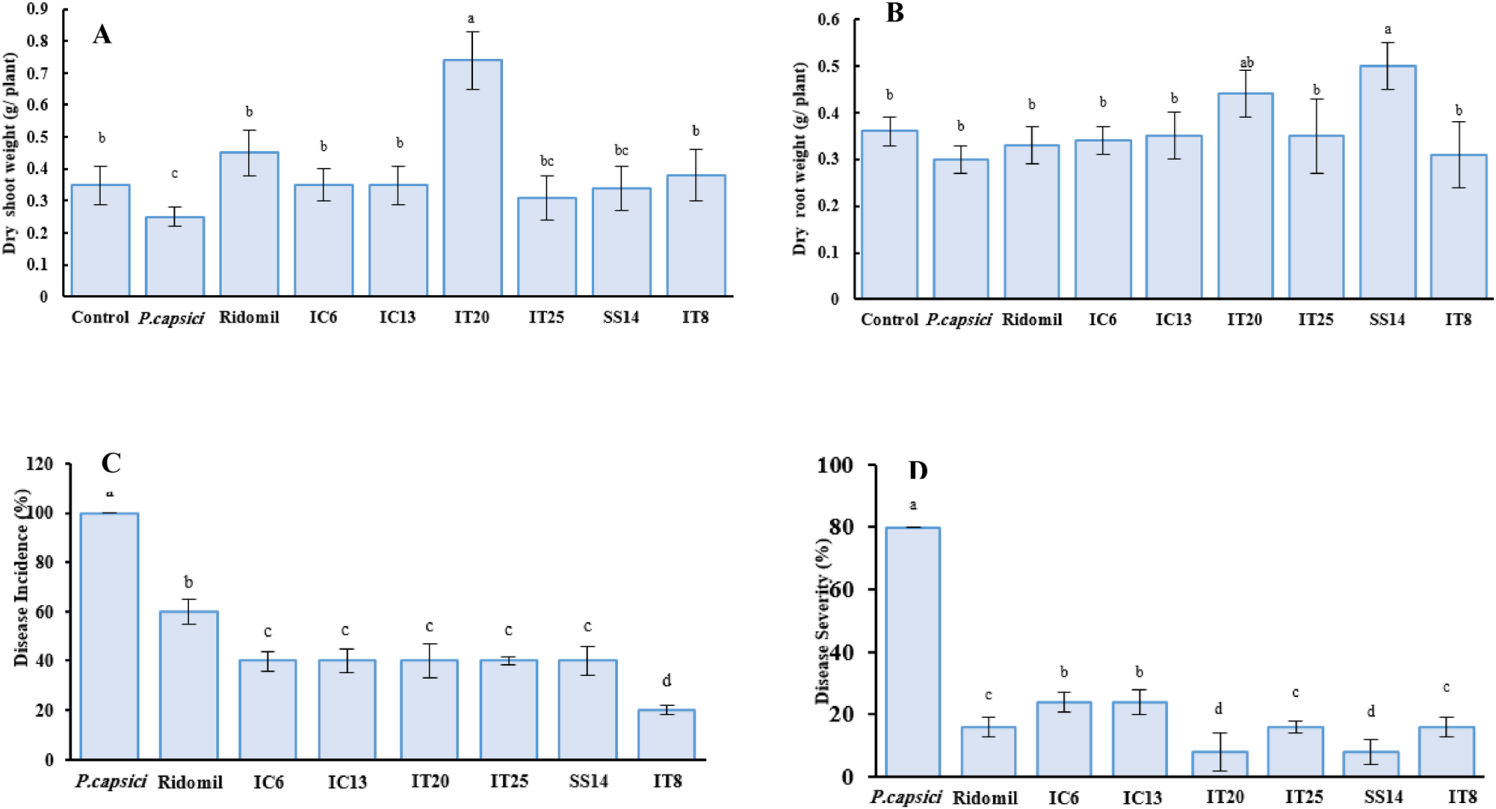
Biocontrol effect of selected antagonistic isolates against pepper blight caused by *Phytophthora capsici* through dry shoot and (**A**) root weight (**B**) disease incidence (**C**) and disease severity (**D**) in greenhouse conditions. Data recorded 15 days after inoculation in seedling stage. Same letters represent non-significant difference according to Duncan’s Multiple Range Test (*P* < *0.05*).

A total of fourteen isolates showed an inhibitory effect against *P. capsici* in dual culture assay. The growth inhibition of the pathogen was different among *Streptomyces* isolates. Six isolates showed an over 40% inhibitory rate. IT20 (69.5%) and SS14 (63.1%) showed the highest percentage of growth inhibition, respectively (Fig. 1S; Table 1). IT20, IT8, SS14 did not have chitinase activity. IC13 and IC6 were able to produce all three examined hydrolytic enzymes (Table 1). The result of in vitro assay indicated a significant positive correlation between protease production and growth inhibition of the pathogen (r = 0.58, *P* < *0.05*). IT20, SS14, and IT8 with cellulase and protease activities, IC13 and IC6 with cellulase, protease, and chitinase activities, IT25 with protease and chitinase activities were selected to be evaluated in the greenhouse experiment.

**Table 1:** In vitro growth inhibition of *Phytophthora capsici* and hydrolytic enzymes production by *Streptomyces* isolates.

Isolate	Growth inhibition (%)	Phosphate solubilizing	Cellulase	Protease	Chitinase
IC6	49.1 ± 0.87^d^*	+	+	+	+
IC10	2.7 ± 0.3^g^	+	−	+	+
IC13	58.7 ± 1.2^c^	+	+	+	+
IS8	18.8 ± 1.1^f^	+	−	+	+
IC15	40.0 ± 0.0^e^	+	−	+	+
SS14	63.1 ± 0.8^b^	−	+	+	−
IT20	69.5 ± 1.0^a^	+	+	+	−
IT8	50.0 ± 0.1^d^	−	+	+	−
IT25	49.6 ± 0.5^d^	+	−	+	+

Values are the means (averaged from three replicates) ± SE.

*Same letters represent non-significant difference according to Duncan’s Multiple Range Test (*P* < *0.05*).

+: Producing −: non-producing.

### Biocontrol potential and growth promotion of the isolates

**Figure 2:**
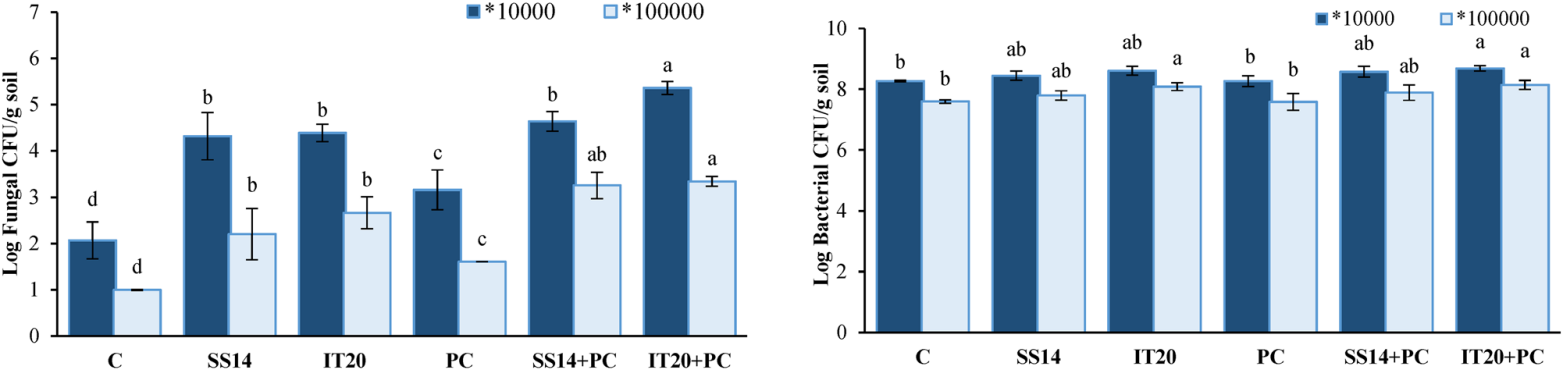
Total fungal colonies (left) on PDA medium supplemented with chloramphenicol (250 ppm) and total bacterial colonies (right) on TSB agar in dilutions 10^–4^ (dark blue) and 10^–5^ (blue) of rhizosphere after 14 days of incubation at 28 ˚C. The same letters represent a non- significant difference according to Duncan’s Multiple Range Test (*P* < *0.05*)**.** PC: *P. capsici*, C: control.

Biocontrol efficacy of the selected isolates against *P. capsici* causing pepper blight disease was evaluated compared to chemical fungicide Ridomil. Minimal dry shoot weight was associated with *P. capsici* (PC). There was a non-significant difference in fresh shoot and root weight among bacterial treatments (data not shown). Dry shoot weight increased in IT20 by 200% and 64% compared to PC and Ridomil respectively (Fig. 1A). SS14 and IT20 significantly increased dry root weight by 66 and 46% compared to PC and Ridomil, respectively (Fig. 1B). All isolates alleviated disease incidence and symptoms into equal or better than Ridomil. The highest level of DI (100%) and DS (80%) was associated with PC. The level of DI in IT8-treated plants was 20% but in the other treatments was not different (Fig. 1C) (F = 2.19; *p* < *0.05*). The level of DS was significantly lower in SS14 and IT20-treated plants (8%). SS14 and IT20 exposed higher disease suppression (10%) than Ridomil (Fig. 1D). In the second part of the greenhouse experiments (non-sterile field soil), IT20 increased disease suppression (19%), shoot length (11%), and dry shoot weight (10%) than SS14 (Fig. 2S) (F = 6.45; *p* < *0.05*).

### Molecular and phenotypical characterizations of the superior isolates

On the medium ISP2, SS14, IT8, and IT20 were differentiated from each other according to the color of spore chains. On ISP3, SS14 and IT20 were distinct according to the color of aerial hyphae. SS14 and IT8 were different from IT20 based on melanin production. Physiological tests showed that IT20 and IT8 had the potential to grow at 42 °C (Table 2). These strains were able to grow on NaCl 6%. Analysis of the 16S rRNA gene sequences showed that IT20 and SS14 were closely related to the genus *Streptomyces* with more than 98% sequence similarity to *S. rochei* and *S. vinaceusdrappus*, respectively (Table 2).

**Table 2:** Phenotypic and molecular characteristics of two selected strains.

Strain	Color of aerial hyphae-spores chains on ISP media			Melanin production	Growth (in/on)			Genbank accession number
	ISP2	ISP3	ISP4	Tyrosine/no Tyrosine media	42 °C	NaCl 6%	NaCl 10%	
SS14	Yellow-dark blue spores	Yellow-dark blue spores	White—grey spores	+**/**+	−	+	−	MH041316
IT20	Yellow-white spores	Cream-light yellow spores	White-grey spores	−**/**−	++	+	−	MK858186
IT8	Yellow-purple spores	Yellow-grey spores	Yellow-purple spores	+**/**+	+	+	−	MG685901

+: presence or growth −: lack or no growth.

### Culturable rhizosphere microbiome

**Figure 3:**
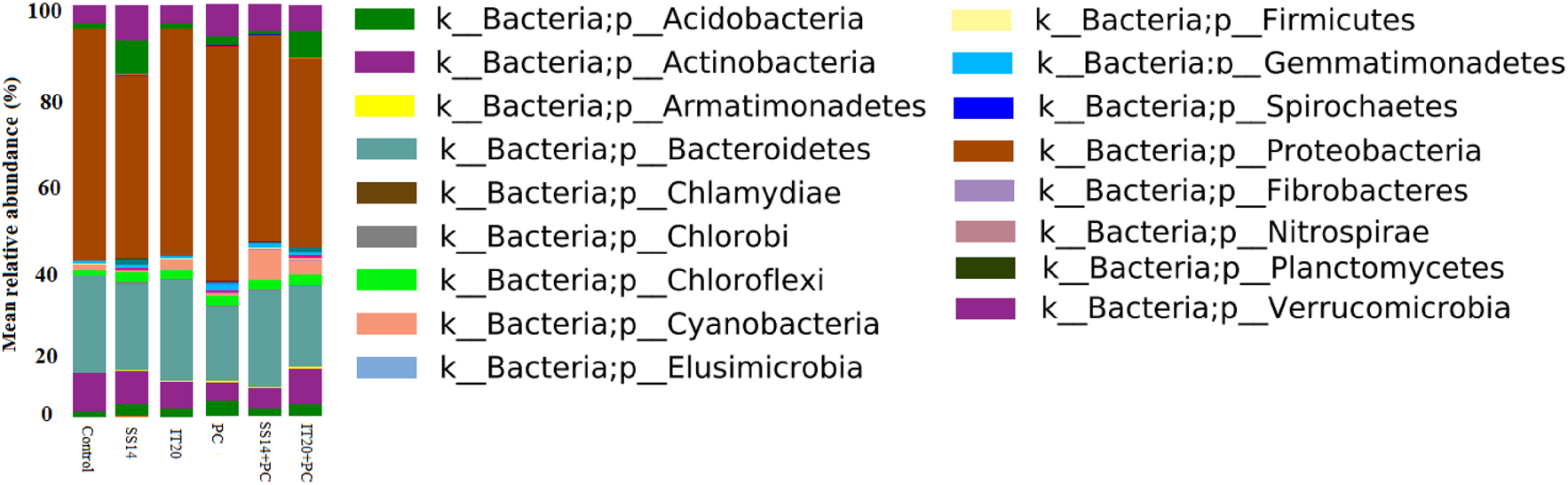
Pepper rhizosphere bacterial community composition in inoculated and non- inoculated samples at the phylum level.

Inoculations of the pathogen and/or *Streptomyces* strains induced changes in the fungal and bacterial colonies. The number of fungal colonies significantly increased in *Streptomyces* treatments compared to control, especially after pathogen inoculation (*P* < *0.05*) (Fig. 2). Pathogen inoculation significantly increased the colonies of *Penicillium* in soil (*P* < *0.05*). In inoculated plants, IT20 caused more variation in culturable soil bacterial colonies than SS14 (Fig. 2S). *Penicillium* type colonies were significantly reduced in SS14 treated-plants (Fig. 3S).

### 16S rRNA gene amplicon Illumina sequencing

A total of 80,355.25 bacterial OTUs were generated from the rhizosphere of six treatments (three replicates per treatment). The number of OTUs generated for IT20, SS14, and control was 16,710 ± 1860, 10,901 ± 1583, and 13,056 ± 1860 respectively. In inoculated plants, the number of OTUs generated for IT20, SS14, and PC was 13,716 ± 2181, 12,577 ± 3455, and 13,695 ± 735 respectively. The bacterial OTUs were associated with 17 phyla, 45 classes, 55 orders, 92 families, and 152 genera. Bacteriome analysis at the phylum level indicated that the rhizosphere of pepper plants was mainly colonized by Proteobacteria (52.2–43.1% of the total sequences), Bacteroidetes (40.3–18.1%), and Actinobacteria (16.0–9.9%) respectively (Fig. 3).

### Diversity and structure of the rhizosphere bacterial communities

**Figure 4:**
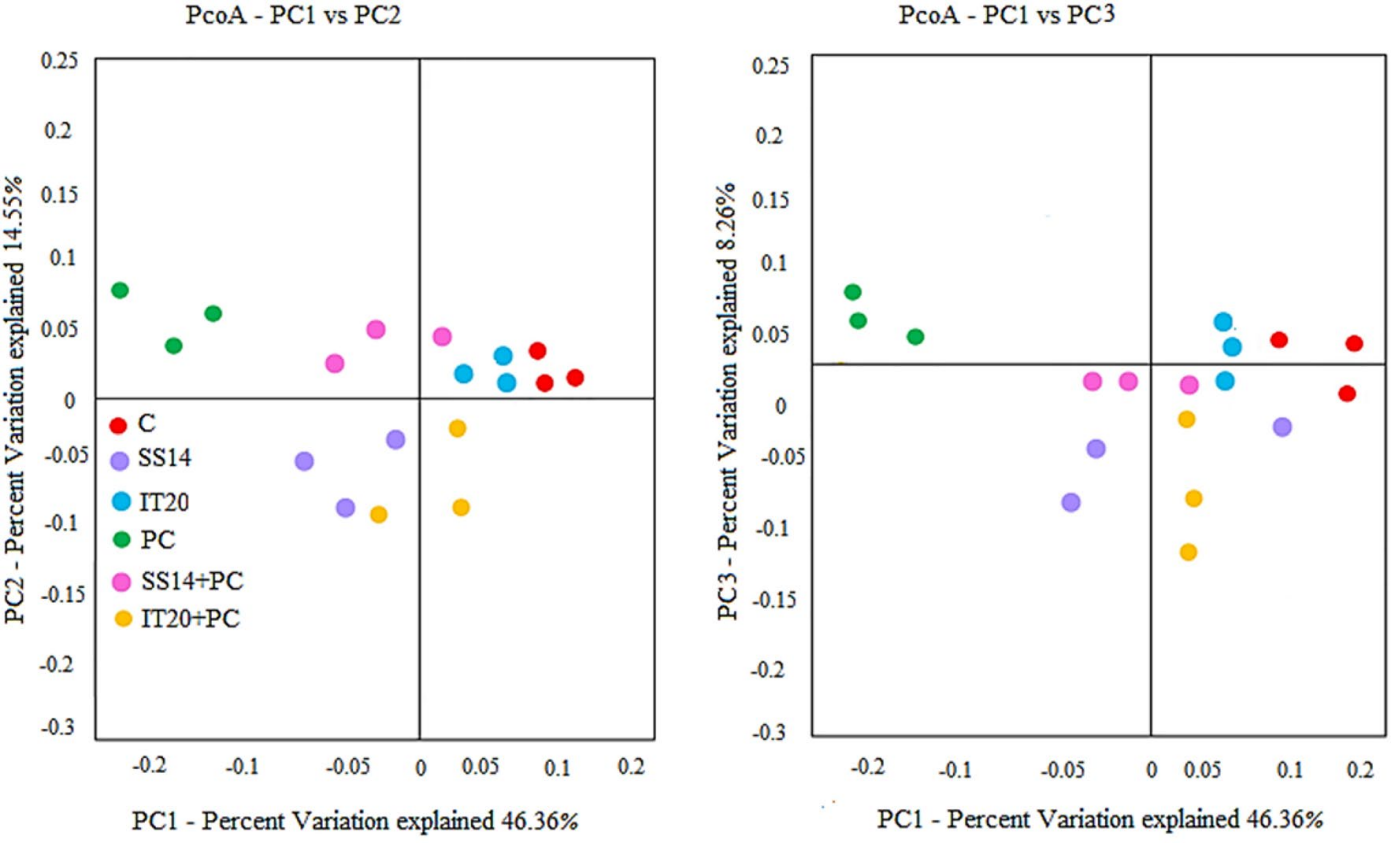
The community clustering is based on Bray–Curtis dissimilarities with weighted UniFrac. Different treatments are indicated with distinct colors.

The levels of microbial diversity were different among the treatments. Alpha rarefaction analysis indicated the phylogenetic diversity tree (PD whole tree), observed species, Shannon, and Simpson reciprocal indices were affected by the bacterial treatments. In inoculated samples, these indices were higher in IT20 than SS14 samples. The dominance index was lower in IT20 inoculated samples (0.007 ± 0.0). Specifically, Chao1 predicted a high value in both inoculated treatments of IT20 (1200.19 ± 20.28) and SS14 (1222.52 ± 40.08) compared to PC (1142.53 ± 5.74). IT20 exhibited a higher alpha-diversity than control samples (Table 3). PERMANOVA (function adonis) found a significant difference among PC and treated plants (PERMANOVA, *p* < 0.01; Fig. 3). Looking at beta-diversity, we found a separated clustering between IT20 and SS14 inoculated with the pathogen in PCoA plots (Fig. 4). By contrast, samples from control and IT20 clustered together (Fig. 4).

**Table 3:** Evaluation of alpha diversity in inoculated and non-inoculated samples.

Treatment	PD whole tree	Chao1	Observed species	Simpson reciprocal
C	66.26 ± 3.63^c^	1083.27 ± 86.3^ab^	851.3 ± 54.42^c^	82.23 ± 18.08^c^
SS14	77.36 ± 7.77^ab^*	1201.65 ± 79.44^ab^	983.46 ± 105.64^ab^	131.47 ± 42.1^ab^
IT20	74.27 ± 1.53^b^	1213.90 ± 30.68^a^	960.0 ± 35.55^b^	104.58 ± 16.50^bc^
PC	76.43 ± 2.18^ab^	1142.53 ± 5.74^b^	930.25 ± 34.05^ab^	109.07 ± 30.28^ab^
SS14 + PC	77.82 ± 3.87^ab^	1222.52 ± 40.08^a^	982.4 ± 54.35^ab^	114.64 ± 42.8^ab^
IT20 + PC	78.22 ± 0.87^a^	1200.19 ± 20.28^a^	1008.58 ± 7.32^ab^	144.88 ± 9.87^a^

Values are the mean counts (averaged from three replicates) ± SE.

*Same letters represent non-significant difference according to Duncan’s Multiple Range Test (*P* < *0.05*).

### Changes of the rhizospheric bacterial community induced by *P. capsici*

The rhizosphere bacteriome of non-inoculated samples was compared with the pathogen inoculated ones. *P. capsici* intensely affected the rhizosphere bacterial community composition. The results revealed that the lowest abundance of Actinobacteria, Bacteroidetes, and Cyanobacteria was associated with PC samples (Fig. 3). Significant changes in the relative abundance of OTUs were identified by Duncan’s Multiple Range under a generalized linear model. The highest relative abundance of *Chitinophaga* (22 ± 0.03%) affiliated with [Saprospirae] was observed in PC (Table 4). PC samples included additional members of Deltaproteobacteria (17.5 ± 0.03%, mostly *Chondromyces*). At the OTU level, Alphaproteobacteria (16 ± 0.03%, 12.1 ± 0%*,* and 6 ± 0% in *Rhodoplanes, Paracoccus,* and *Asticcacaulis* respectively), Flavobacteriia (9.7 ± 0.05%, *Flectobacillus*), Betaproteobacteria (8.7 ± 0.03%, *Massilia*), Gammaproteobacteria (11.5 ± 0.1% and 6.7 ± 0.05% in *Rhodanobacter* and *Hydrocarboniphaga,* respectively), Acidobacteriia (*Granulicella*, 8.8 ± 0.14%), and Gemmatimonadetes (2.7 ± 0.01%, *Gemmatimonas*) profited from the rhizosphere of the diseased plants. In contrast, Gammaproteobacteria (0.3%, *Thermomonas)* and Bacilli (0.7%, *Luteolibacter)* was lower in PC compared to other samples (Table 4).

**Table 4:** Influence of *P. capsici* on the prokaryotic community composition of pepper rhizosphere.

OUT	Class	Genus	Control	SS14	IT20	PC	SS14 + PC	IT20 + PC
OTU-8495	Acidobacteriia	*Granulicella*	0 ± 0^a^*	18.6 ± 0.3^f^	4 ± 0.03^c^	8.8 ± 0.14^de^	10 ± 0.1^g^	4 ± 0.04^d^
OTU-5061	[Saprospirae]	*Chitinophaga*	0 ± 0^a^	0 ± 0^a^	0 ± 0^a^	22 ± 0.04^j^	1 ± 0^ab^	0 ± 0^a^
OTU-15411	Flavobacteriia	*Segetibacter*	0.5 ± 0^a^	3.1 ± 0.2^bc^	2.8 ± 0.03^b^	1.4 ± 0^ab^	4.6 ± 0.1^d^	1.5 ± 0.01^ab^
OTU-1559		*Flectobacillus*	1 ± 0.01^ab^	7.5 ± 0.04^d^	1.3 ± 0.01^ab^	9.7 ± 0.05^e^	3.5 ± 0.02^bc^	2.3 ± 0^b^
OTU-190	Cytophagia	*Larkinella*	0.3 ± 0^a^	0.7 ± 0^a^	0.2 ± 0^a^	2.3 ± 0.01^b^	0.3 ± 0^a^	0.4 ± 0^a^
OTU-849	Alphaproteobacteria	*Caulobacter*	0.5 ± 0^a^	0.2 ± 0^a^	0.6 ± 0^a^	3.6 ± 0.04^c^	3.3 ± 0.03^bc^	1.6 ± 0.01^ab^
OTU-368		*Asticcacaulis*	3 ± 0.01^b^	3.5 ± 0^c^	1.1 ± 0^ab^	6 ± 0^c^	1.3 ± 0.01^ab^	3.4 ± 0.01^cd^
OTU-2070		*Parvibaculum*	0.3 ± 0^a^	1.6 ± 0.02^ab^	0.8 ± 0^a^	1.7 ± 0.01^ab^	0.2 ± 0^a^	1 ± 0.01^a^
OTU-3993		*Altererythrobacter*	0.6 ± 0^a^	0 ± 0^a^	0.5 ± 0^a^	2.4 ± 0^b^	0.3 ± 0^a^	1.1 ± 0.01^a^
OTU-20358		*Paracoccus*	6 ± 0.02^d^	4.4 ± 0.04 ^c^	1.8 ± 0.01^ab^	12.1 ± 0^f^	6.5 ± 0.02^de^	4.1 ± 0.02^d^
OTU-1300		*Hyphomicrobium*	0 ± 0^a^	0 ± 0^a^	0.1 ± 0^a^	1.6 ± 0.01^ab^	0.4 ± 0.01^a^	0.5 ± 0^a^
OTU-2183		*Rhodoplanes*	4.3 ± 0.01^c^	10 ± 0.08^e^	8.6 ± 0.05^d^	16 ± 0.03^ g^	14.5 ± 0.06^h^	10 ± 0.04^e^
OTU-70	Betaproteobacteria	*Massilia*	0.2 ± 0^a^	0.7 ± 0^a^	4.2 ± 0.04^c^	8.7 ± 0.03^de^	4 ± 0.02^cd^	3.2 ± 0.01^cd^
OTU-16343		*Ramlibacter*	1.9 ± 0.02^ab^	4.5 ± 0.04 ^c^	0.6 ± 0^a^	1.3 ± 0^ab^	1 ± 0.01^ab^	0.4 ± 0^a^
OTU23068	Gammaproteobacteria	*Thermomonas*	11.5 ± 0.13^e^	2.4 ± 0^b^	14.6 ± 0.1^e^	0.3 ± 0^a^	7 ± 0.03^f^	3 ± 0.01^cd^
OTU-24		*Rhodanobacter*	0.8 ± 0.01^a^	2.2 ± 0.02^b^	0.5 ± 0^a^	11.5 ± 0.1^f^	0.8 ± 0.03^a^	0.5 ± 0^a^
OTU-16335		*Hydrocarboniphaga*	0 ± 0^a^	0 ± 0^a^	0.4 ± 0^a^	6.7 ± 0.05^d^	0 ± 0^a^	0.8 ± 0^a^
OTU-192		*Stenotrophomonas*	0 ± 0^a^	2.5 ± 0.1^b^	0.2 ± 0^a^	2.1 ± 0.02^b^	0.4 ± 0^a^	0.2 ± 0^a^
OTU-5010	Deltaproteobacteria Verrucomicrobia	*Chondromyces*	2.9 ± 0.01^b^	0.7 ± 0.01^a^	2.8 ± 0.01^b^	17.3 ± 0.03h	3.7 ± 0^bcd^	0.8 ± 0.01^a^
OTU-10973		*Opitutus*	0.07 ± 0^a^	0.2 ± 0^a^	0.8 ± 0^a^	0.4 ± 0^a^	1.5 ± 0.01^ab^	2 ± 0.01^bc^
OTU-185		*Prosthecobacter*	0 ± 0^a^	0 ± 0^a^	0 ± 0^a^	1.3 ± 0^ab^	2.4 ± 0.01^b^	0.7 ± 0^a^
OTU-205		*Prosthecobacter*	0 ± 0^a^	0.5 ± 0^a^	0.4 ± 0^a^	1.5 ± 0.01^ab^	0.6 ± 0^a^	0.4 ± 0.01^a^
OTU-1330	Gemmatimonadetes	*Gemmatimonas*	0.3 ± 0^a^	0.4 ± 0^a^	0 ± 0^a^	2.4 ± 0.02^b^	1 ± 0^a^	0.7 ± 0^a^
OTU-602	Bacilli	*Peanibacillus*	1.7 ± 0^ab^	1.8 ± 0.01^ab^	0.2 ± 0.01^a^	2.7 ± 0.01^b^	1.1 ± 0.01^ab^	1.8 ± 0^ab^
OTU-554		*Sporosarcina*	1.9 ± 0^ab^	1.5 ± 0^ab^	0.8 ± 0^a^	3.2 ± 0.01^bc^	3.1 ± 0.03^bc^	1 ± 0.01^a^
OTU-30547		*Luteolibacter*	3.6 ± 0.02^bc^	1.5 ± 0.02^ab^	0.5 ± 0^a^	0.7 ± 0^a^	1.4 ± 0.01^ab^	0.4 ± 0^a^

Values are the mean percentages (averaged from three replicates) ± SE.

*Same letters represent non-significant difference according to Duncan’s Multiple Range under a generalized linear model (GLM) (*P* < *0.05*).

### Changes of the rhizospheric bacterial community induced by *Streptomyces* strains

**Figure 5:**
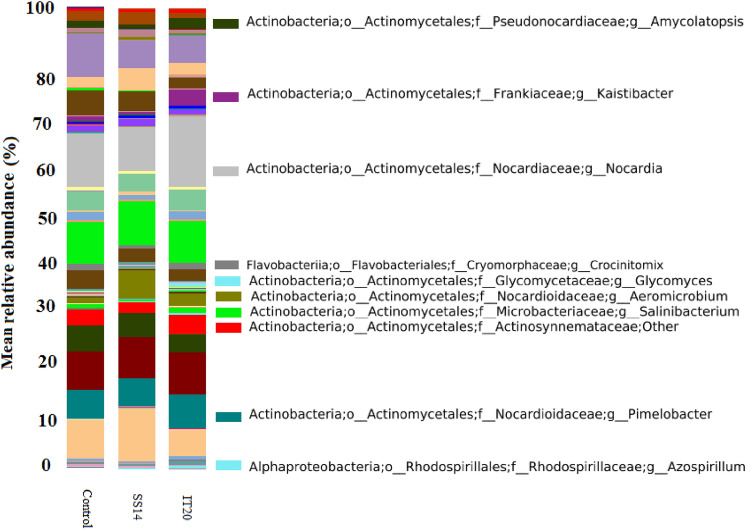
Effects of IT20 or SS14 on the pepper rhizospheric bacterial community at the genus level.

The rhizosphere bacteriome of two *Streptomyces* treated-plants was compared with control plants. The results showed that the relative abundance of Cyanobacteria increased in IT20 (72%) compared to SS14 and control (47%) samples (t-test, *p* < 0.05; Fig. 5). The abundances of OTUs *Kaistibacter* (75.5%), *Glycomyces* (66%), *Amycolatopsis* (65%), *Nocardia* (51%), and *Salinibacterium* (49%) affiliated with Actinobacteria, *Crocinitomix* (50%), and *Azospirillum* (50%), respectively affiliated with Flavobacteriia and Alphaproteobacteria were significantly enriched in IT20 compared to SS14. In contrast, *Aeromicrobiom* affiliated with Actinobacteria increased (60%) in SS14 compared to IT20.

### Changes in the rhizospheric bacterial community modulated by the interaction *Streptomyces *strains with *P. capsici*

The differences among inoculated samples were distinct at the phylum level (Fig. 3). In inoculated IT20 samples, the members of Planctomycetes (10%) and Actinobacteria (9%) exhibited a high relative abundance among other phyla. The relative abundance of Cyanobacteria increased in both SS14 (100%) and IT20 (74%) compared to PC samples. In contrast, the relative abundance of Gemmatimonadetes decreased in both SS14 (43%) and IT20 (20%) compared to PC samples. Bacteroidetes, particularly Flavobacteria (*Fluviicola*), exposed a higher relative abundance in IT20 (9.4 ± 0.03%) than SS14 (3.9 ± 0.01%). While *Larkinella* significantly decreased in both SS14 (0.3) and IT20 (0.4) compared to PC (2.3). OTUs affiliated to *Sporocytophaga, Dyadobacter, Polaromonas, Arenimonas, Pseudomonas, Cellvibrio,* and *Mycoplana* indicated a high relative abundance in both IT20 and SS14, while *Achromobacter, Dokdonella, Lysobacter,* and *Sphingobium* were enriched only in IT20 samples (Table 5). The highest relative abundance was observed in OTUs *Devosia* affiliated with Gammaproteobacteria (60 ± 0.04%). The relative abundance of *Gallionella* significantly increased in IT20 (26.5 ± 0.3%) compared to SS14 (7.7 ± 0.1%), while *Rhizobium* increased only in SS14 samples (20.6 ± 0.19%). *Caulobacter*, *Altererythrobacter*, and *Stenotrophomonas* affiliated to Proteobacteria were shared between IT20 and SS14 treatments. OTUs *Sporosarcina* affiliated with Bacilli indicated a three-fold decrease in IT20 compared to SS14 and PC samples (Table 5).

**Table 5:** Interaction among *P. capsici*, *Streptomyces* strains (SS14 and IT20), and native rhizosphere bacteriome.

OUT	Class	Genus	Putative function	PC	SS14 + PC	IT20 + PC		
OTU-975	Flavobacteria	*Fluviicola*	Active root colonizing^18^	2.5 ± 0.01^b^*	3.9 ± 0.01^cd^	9.4 ± 0.03^d^		
OTU-1314		*Parasegitibacter*	–	1.0 ± 0^ab^	0.8 ± 0^a^	2.4 ± 0.03^ab^		
OTU-242	Sphingobacteriia	*Pedobacter*	Mutualist with nematodes^19^	0 ± 0^a^	0.3 ± 0^a^	0.7 ± 0^a^		
OTU-4562		*Sporocytophaga*	–	0 ± 0^a^	2.4 ± 0.01^ab^	2.4 ± 0.0^ab^		
OTU-174	Cytophagia	*Algoriphagus*	–	0.6 ± 0^a^	0.8 ± 0^a^	1.9 ± 0^ab^		
OTU-3173		*Rhodocytophaga*	Growth promoting^20^	0 ± 0^a^	0.1 ± 0^a^	0.6 ± 0^a^		
OTU-71		*Dyadobacter*	Biocontrol^21^	0.7 ± 0 ^a^	7.29 ± 0.03^e^	8.6 ± 0.08^cd^		
OTU-1279	Betaproteobacteria	*Pigmentiphaga*	–	2.4 ± 0^b^	1.8 ± 0^ab^	3.3 ± 0^b^		
OTU-695		*Achromobacter*	Wilt disease suppression^22^	0 ± 0^a^	1.7 ± 0.05^ab^	8.1 ± 0.01^cd^		
OTU-6001		*Gallionella*	Wheat plant healthy^23^	0.3 ± 0 ^a^	7.7 ± 0.1^e^	26.5 ± 0.3^e^	7.7	26.2
OTU-520		*Polaromonas*	–	0 ± 0^a^	5 ± 0.02^d^	5.3 ± 0.01^bc^		
OTU-20333		*Janthinobacterium*	Antimicrobial activity^24^	0.3 ± 0 ^a^	0.3 ± 0 ^a^	2 ± 0.02^ab^		
OTU-41		*Methylotenera*	Active rhizosphere bacteria^25^	0.3 ± 0 ^a^	1.4 ± 0.02^ab^	2.2 ± 0.01^ab^		
OTU-444	Gammaproteobacteria	*Arenimonas*	Bio-fertilizer^26^	23.5 ± 0.13^d^	40 ± 0.2^ g^	50 ± 0.01^f^		
OTU-17904		*Aquicella*	Banana plant healthy^27^	4.5 ± 0.01^c^	3.6 ± 0.01^cd^	8.4 ± 0^d^		
OTU-1247		*Dokdonella*	Active root colonizing^18^	0 ± 0^a^	0 ± 0^a^	7.1 ± 0.0^cd^		
OTU-4910		*Pseudomonas*	Biocontrol^28^	0.3 ± 0^a^	2 ± 0.01^ab^	5 ± 0.04^bc^		
OTU-18008		*Cellvibrio*	Biocontrol^29^	0.67 ± 0^a^	3 ± 0.03^bc^	6 ± 0.07^c^		
OTU-1955		*Lysobacter*	Biocontrol of *Phytophthora* sp.^30^	0 ± 0^a^	0 ± 0^a^	2 ± 0^ab^		
OTU-124		*Pseudoxanthomonas*	Active rhizosphere bacteria^31^	0 ± 0^a^	0.5 ± 0^a^	0.3 ± 0^a^		
OTU-29	Alphaproteobacteria	*Devosia*	Biocontrol^32^	0 ± 0^a^	3.7 ± 0.01^cd^	60 ± 0.04^g^		
OTU-293		*Methylobacterium*	–	0.9 ± 0.01^ab^	1.5 ± 0.01^ab^	1.6 ± 0^ab^		
OTU-1506		*Phaeospirillum*	Endophyte^33^	2.4 ± 0.01^b^	2.5 ± 0.01^b^	3.5 ± 0^b^	0.17	0.4
OTU-452		*Mycoplana*	IAA-producing^34^	0 ± 0^a^	2.6 ± 0.03^b^	2 ± 0^ab^	0.34	0.28
OTU-5822		*Rhizobium*	IAA-producing^35^	0 ± 0^a^	20.6 ± 0.19f	1 ± 0.01^a^		
OTU-4		*Agrobacterium*	Growth promoting endophyte^36^	1.0 ± 0.01^ab^	1.2 ± 0.01^a^	2 ± 0.0^ab^		
OTU-20275		*Sphingobium*	Biocontrol^37^	0 ± 0^a^	0 ± 0^a^	2 ± 0^ab^		
OTU-10641		*Parvibaculum*	–	0.3 ± 0.01^a^	1.5 ± 0.02^ab^	1.5 ± 0^ab^		
OTU-1283	Verrucomicrobiae	*Verrucomicrobium*	Carbon cycling endophyte^38^	0 ± 0^a^	0.8 ± 0^a^	1.4 ± 0.01^ab^		

Values are the mean percentages (averaged from three replicates) ± SE.

*Same letters represent non-significant difference according to Duncan’s Multiple Range under a generalized linear model (GLM) (*P* < *0.05*).

### Strain-specific assembly of Actinobacteria and correlation analysis

**Figure 6:**
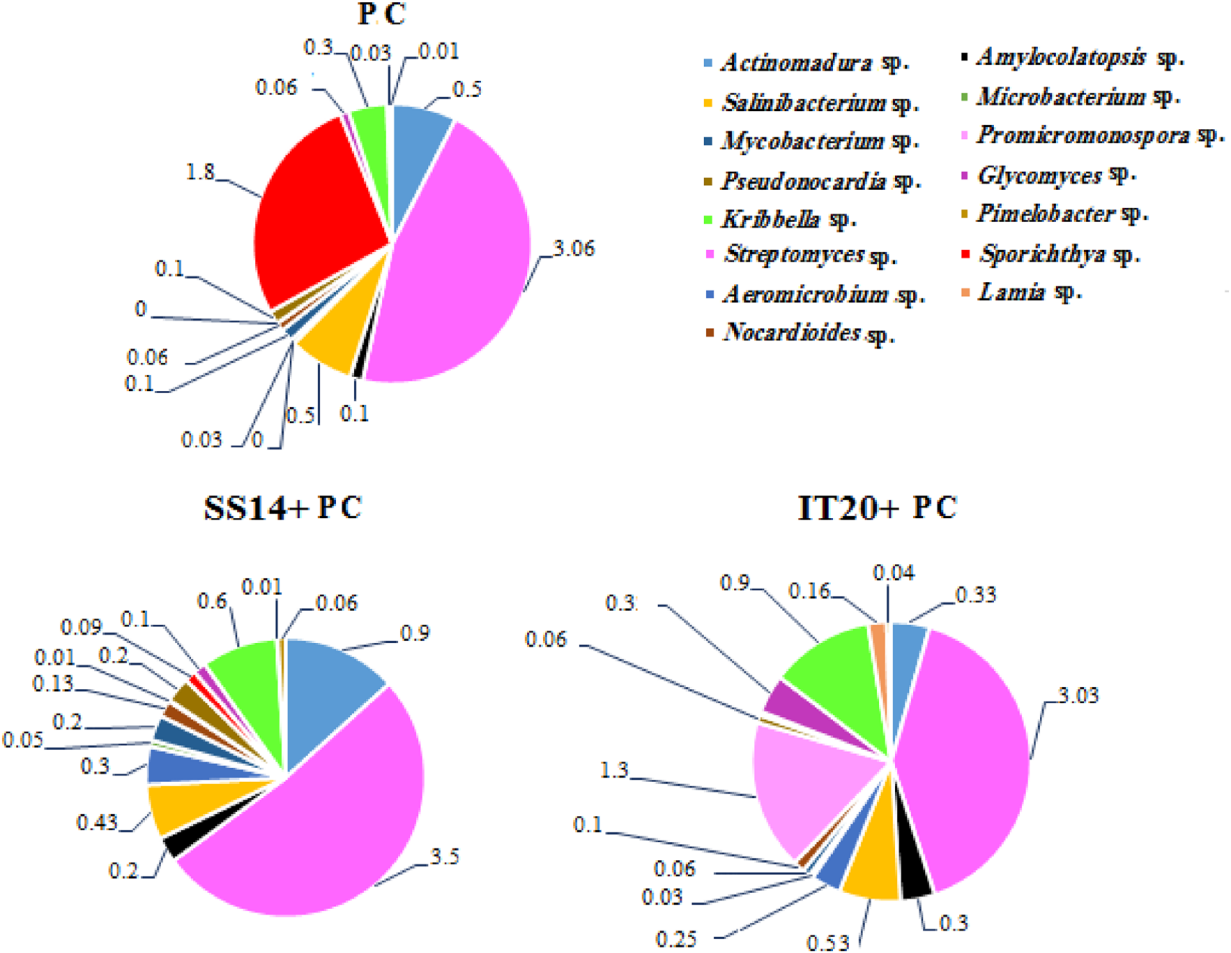
Mean relative abundance (transformed in %) of the genera of Actinobacteria in soil prokaryotic community in the inoculated bacterial treated plants (IT20 and SS14) or non-treated (PC). The average value of three replicates is reported for each sample.

Inoculation of pathogen modulated specific changes in the community of Actinobacteria. Strain-specific changes were evident when IT20 and SS14 treatments were compared. The differences were distinguishable at the genus level (Fig. 6). Two OTUs (*Aeromicrobium* and *Promicromonospora*) were unique for IT20 while one OTU (*Sporichthya*) was unique for PC. Both treatments of IT20 and SS14 increased diversity and changed the occurrence and abundance of the genera of Actinobacteria. The abundances of phylotypes including *Promicromonospora* (129%), *Kribbella* (30%), *Lamia* (15%), *Amylocolatopsis* (10%), and *Salinibacterium* (10%) genera increased in IT20 samples compared to SS14 (Fig. 6)*.* As shown in Table 6, there was a significant positive correlation between the occurrence of *Promicromonospora* and *Devosia* with disease suppression (*p* < *0.01*). Also, there was a positive correlation between the co-occurrence of *Promicromonospora* with *Kribbella* (r = 0.801), *Amylocolatopsis* (r = 0.685), *Microbacterium* (r = 0.670)*, Nocardioides* (r = 0.625), and *Aeromicrobium* (r = 0.605). Besides, there was a significant positive correlation between the occurrence of *Sphingomonas* with *Actinomadura* (r = 1) and *Streptomyces* (r = 0.865) (*p* < *0.01*)*.* Moreover, there was a positive correlation between the co-occurrence *Streptomyces* with *Pseudonocardia* (r = 1), *Nocardioides* (*r* = 0.745), *Mycobacterium* (*r* = 0.718), and *Microbacterium* (r = 0.694)*.* In contrast, a significant negative correlation was observed between the occurrence of *Nocardioides* (r = − 0.840) with *Sporichthya* (*p* < *0.01*). Also, there was a significant negative correlation between the occurrence of *Sporichthya* with *Pseudonocardia* (r = − 0.694), *Microbacterium* (r = − 0.622)*,* and *Pseudomonas* (r = − 0.607) (Table 6).

**Table 6:** Correlation analysis among occurrence of the genera of Actinobacteria with different genera in disease suppression/prevalence.

Genus	Disease suppression	Disease prevalence	*Sporichthya*	*Kribbella*	*Promicromonospora*	*Microbacterium*	*Streptomyces*	*Actinomadura*
*Microbacterium*	0.675*		− 0.622*	0.670*	0.718**		0.694*	0.833**
*Promicromonospora*	1**			0.801**		0.718**		
*Nocardioides*			− 0.840**		0.625*	0.706**	0.745**	0.785**
*Mycobacterium*			− 0.694*	0.625*		0.635*	0.718**	0.710**
*Pseudonocardia*							1**	
*Aeromicrobium*					0.605*			
*Amylocolatopsis*				0.725**	0.685*			
*Erythromicrobium*	0.621*							
*Pseudomonas*			− 0.607*	0.620*				
*Devosia*	0.905**				0.680*			
*Rickettsia*		1**						
*Niastella*		1**						
*Sphingomonas*							0.865**	1**

Significant effects are shown as *p < *0.05* and **p < *0.01.*

## Discussion

The activity of hydrolytic enzymes is one of the effective mechanisms of *Streptomyces* to inhibit fungal growth^2,39^. Our results are following a recent study^40^ that showed the protease activity of *Paenibacillus polymyxa* is also involved in the antagonism against *Phytophthora*.

In this study, two strains *S. vinaceusdrappus* SS14 and *S. rochei* IT20 were different in terms of P solubilizing ability and melanin production. There is growing evidence that rhizosphere–microbe interactions are modulated by nutrient availability in the soil that bacterial communities act somewhat independently of plants^41^. In particular, the concentration of available P in the soil adjusted the establishment of them^42^ or induced plant immunity through *PR1* and *WRKY40* gene expression which induced salicylic acid‐dependent responses improving resistance against the pathogen^43^. Recent studies also showed that plants with a superior defense and nutrient acquisition, possibly, have specific microbial communities in the rhizosphere, suggesting a close link between plant growth parameters and rhizosphere microbiota functions. A fine example was recently provided that P starvation response 1 (PHR1) in *Arabidopsis* regulates P stress responses regulated a functionally appropriate set of immune-related genes and contributed to the assembly of root microbiomes^44^.

About 40% of *Streptomyces* species produce melanin pigments, dark-brown to black, on tyrosine-containing agar media^45^ that are not essential for the growth and development of them but play a vital role in their survival and competitiveness. They have multiple functions including antioxidant and antimicrobial activities, tolerance to extreme conditions, and UV radio-protective^45^ to prepare light stable bio-pesticides^46^. Conversely, melanin production and tyrosinase activity of soil bacteria (e.g. *Rhizobium* and *Azospirillum*) play a role in their symbiotic relationship with plants^45^. In this study, a melanin-producing strain SS14 suppressed the frequency of some *Penicillium* type colonies and the relative abundance of some bacterial OTUs in the rhizosphere (Fig. 2). Some species of *Penicillium* have P solubilizing ability and are involved in plant growth promotion^47^. Interestingly, SS14 increased the abundance of *Rhizobium* indicating the responses of *Rhizobium* to biocontrol species of *Streptomyces* is depending on bacterial strains. To our knowledge, this is the first to report the cooperative interactions between melanin-producing strain *Streptomyces* and *Rhizobium* under the biotic stress condition. Under the non-sterile condition, IT20 showed an increased capacity to suppress *Phytophthora* blight and promote plant growth. The higher number of total bacterial and fungal colonies associated with IT20 may be correlated with the increased plant biomass which was not observed for SS14. Illumina amplicon sequencing analysis of 16S rRNA gene revealed that IT20 differently manipulated soil prokaryote communities compared to SS14. IT20 had higher microbial community responders than the melanin-producing strain SS14. Therefore, inorganic P solubilizing capacity and P compounds have significant effects in interactions between soil beneficial microbes*.* Hence, melanin production provided less priority than P solubilizing to select biocontrol strains of *Streptomyces* in the soil applications.

Community differences between IT20 and control samples were less pronounced but phylum Cyanobacteria was differentially abundant. The community of Cyanobacteria improve soil fertility through nitrogen fixation and balancing mineral nutrition in the soil. Many members of cyanobacteria are known to release various kinds of biologically active components like phytohormones that act as an elicitor to promote plant growth^48^. Hence, increasing growth parameters like shoot length and plant biomass had been exposed to be positively linked with the various phytochemical components promoted by Cyanobacteria^48^.

The prokaryotic communities shaped with phylogenetically diverse OTUs that relative abundance of them increased or decreased compared to PC (Table 4). There is a positive correlation in the interaction between *Streptomyces* strains with rhizospheric bacteria that resulted in a lower abundance of some OTUs and lower disease prevalence (Tables 5, 6). Some phylotypes of these genera could correspond with pathogenic interactions or respond to pathogen inoculation. The different mechanisms could increase the abundance of specific plant-associated microbes. The stress condition modulates the root exudate secretion, which consequently attracts specific microbes. For example, *Arabidopsis* roots in response to a leaf pathogen infection attract *Bacillus subtilis* into the rhizosphere^49^. In the current study, the most enrichment in IT20 samples was recorded for the genus *Devosia* affiliated to Gammaproteobacteria, it was previously described that this genus is increased in response to *B. velezensis* and *P. fluorescens*, involved in biocontrol activity against *R. solanacearum* on tomato^50^. Another increase in relative abundance was recorded for the genus *Gallionella* that was previously described as a member of the core microbiome of the wheat healthy plant^23^. The plant–microbe interactions and plant ability to select neighbors may potentially benefit the plant's growth or defense^32^. The abundance of beneficial microbes is enriched to compete for space and resources using antimicrobial compounds that prevent pathogen growth and virulence^51^. Similar trends were observed for *Dokdonella* and *Sphingobium* that were enriched. Some strains of *Sphingomonas* produce indole acetic acid (IAA) and have protective effects that could be a member of the microbiome in disease-suppressive soils^52^. *Dokdonella* is an aerobic, non-spore-forming, gram-negative soil bacteria reported as an active root colonizing agent^18^. The other enrichment was observed in *Achromobacter*. As previously reported, *A. xylosoxydans* exhibited an antifungal effect and significantly reduced *Fusarium* wilt disease of tomato plants^22^. Therefore, this reveals that the population of some rhizospheric bacteria can increase as a response to different soil-borne fungal pathogens. The responders shared between both *Streptomyces* strains IT20 and SS14 (Table 5) shape the microbiota to inhibit pathogen growth, which consequently ameliorated disease suppression.

There were notable differences in the community pattern of Actinobacteria among inoculated samples. Spearman’s rank correlation coefficient showed a clear positive correlation in the interaction of IT20 with the members of Actinobacteria resulted in a higher abundance of corresponding OTUs and lower disease severity (Table 6). These bacteria might play beneficial roles in pepper plants such as supplying nutrients, conferring resistance against pathogens, and anti-oomycete. In contrast, OTUs affiliated to Bacilli including *Peanibacillus*, *Sporosarcina*, and *Luteolibacter* decreased in two *Streptomyces* treatments. A similar trend was reported by Araujo et al.^53^ indicating that the application of biocontrol *Streptomyces* strains promoted wheat plant growth and modulated the root microbiome by decreasing *Paenibacillus* and increasing other beneficial bacterial OTUs. Interestingly, Guo et al.^54^ applied a consortium of three PGPR strains (*B. cereus*, *B. subtilis*, and *Serratia* sp.) to suppress *Phytophthora* blight disease resulted in a negative association between *Phytophthora* disease prevalence and the relative abundance of *Sporichthya*. Therefore, these results indicate shifting in bacterial community composition induced by biocontrol species of *Bacillus* could be different from *Streptomyces* strains to suppress the same pathogen. Therefore, this proposes a possible cross-talk pathway that occurs between bacterial biocontrol agents to manipulate and shape the microbiome. Most importantly, *Sporichthya* was closely suppressed under the presence of IT20, undoubtedly proving the antagonistic mode of interaction between IT20 and *Sporichthya* (Fig. 6).

The co-occurrence of microbes is linked to nutritional interrelationship^55^. In this situation, metabolites of one microbe can be utilized by other community members, then can cause a higher enrichment of microbial species in response to the pathogen^56^. The community pattern of Proteobacteria and Actinobacteria displayed different relationships with two *Streptomyces* strains and disease suppression. Cooperative relationships are the selective perceptions driving specific rhizospheric bacterial assemblages with plants^57^. The occurrence of these bacteria is being for the first time reported against *P. capsici*.

Our current understanding of microbiota-mediated plant protection provides an opportunity to recognize and characterize the positive plant microbial interactions for plant growth and survival under stress conditions. The potent biocontrol taxa, helper communities predicted through correlation analysis, would allow designing and constructing synthetic microbial communities (SynComs) for developing efficient inoculants. Overall, constructed communities provide a model to hypothesize and optimize targeted plant disease management and plant growth promotion. A better understanding of the microbiome between plant species and genotypes will increase our ability to efficiently manipulate plant–microbe systems for stable and predictable results in the open fields.

## Conclusion

Plant growth-promoting *Streptomyces* species are used as natural alternatives to synthetic fungicides. Using high throughput sequencing method and microbiome profiling for the first time the dynamics of rhizosphere bacterial communities manipulated by phosphate solubilizing *Streptomyces* strain was explored and correlated with higher plant growth promotion and disease suppression. Actinobacteria were enriched following pathogen inoculation. In addition to enzyme activities, investigations on other characteristics of superior biocontrol strains of *Streptomyces* such as secondary metabolite profile and how they impact the assembly of the rhizospheric bacterial communities subsequent pathogen attack could be valuable to optimally design and develop SynComs of Actinobacteria for improving agricultural productivity and environmental sustainability.

## Materials and methods

### Microorganisms

Fourteen isolates were selected from the Agricultural Biotechnology Research Institute of Iran Culture collection (ABRIICC) based on the plant growth-promoting (PGP) and antifungal activities^2^. PGP traits including siderophore production, phosphate solubilizing ability, indole-3-acetic acid production, and enzyme activities including chitinase, protease, and cellulase were evaluated in a previous study^2^. The *Oomycete* pathogen (*P. capsici* ABRIICC 10292) was provided by ABRIICC and the pathogenicity test was conducted using plug inoculation on pepper seedlings (data not shown).

### Antagonistic effect of isolates

The bacterial suspension of each isolate (20 μL of a 10^8^ CFU/mL sterile saline solution) was cultured linearly on the two opposite sides (1 cm from the plate edge) of potato dextrose agar (PDA) plates and incubated at 29 °C for 48 h. Then, one fungal plug (0.5 cm diameter) was placed at the center of each plate^58^. Plates incubated at 29 °C for 4 days. The percent of growth inhibition was calculated using the formula [(x − y)/x × 100], where ‘x’ is the fungal growth radius of a control culture (in cm) and ‘y’ is the distance of the pathogen growth in the direction of bacteria (in cm). Data obtained from in vitro experiments reported the average value of three biological replicates ± SE.

### Biocontrol potential of the selected isolates and soil sampling

For the first experiment, sterilized seeds of bell pepper (*Capsicum annuum* L. cv 9325 seminis) were placed into pots (10 × 15 cm) filled with sterile field soil and peat moss (2:1 v/v), with one seedling occupying each cell. Seedlings were watered every two days with tap water and kept in a greenhouse at 27 °C and 16 h brightness/8 h darkness. Bacterial treatments (*Streptomyces* cell and spores) were prepared according to a previous study^2^. Five gram of sand containing bacteria was added to the surface of each cultivated pot. Sterilized sand was used as a control. After 7 days of treatment (for the establishment of bacteria), plants were inoculated with the plugs (2 × 2 cm^2^) of the 5 days-old *P. capsici* at a distance of 1 cm from the crown of each plant. The air temperature varied from 22 to 28 °C during the trial. The treatments including, control (mock inoculation), positive control (*P. capsici*), Ridomil (soil drenched with fungicide in a concentration of 1.5 g/L), and six *Streptomyces* isolates (IC6, IC13, IT20, IT25, SS14, and IT8) into inoculated or non-inoculated pathogen. The greenhouse experiment was carried out in randomized blocks design with five blocks with five biological replicates for each treatment.

For the second experiment, the seedlings were placed in pots (15 × 20 cm) filled with a mixture of non-sterile field soil (bulk soil) and peat moss (2:1 v/v). Two selected strains (IT20 and SS14) were evaluated into pathogen inoculated or non-inoculated treatments compared to control (C) and positive control (PC) with five replicates. Rhizosphere was sampled from each pot. After 15 days of inoculation, the plants were harvested and plant traits (shoot length, shoot, root fresh, and dry weight) were measured. Disease incidence (DI), disease severity (DS), and disease suppression (1- DS) were assessed. DS was conducted on a scale from 0 to 5: 0 = no symptoms = 0%, 1 = leaf yellowing = 25%, 2 = minor stem necrosis = 50%, 3 = moderate stem necrosis and some leaf wilt = 75%, 4 = severe stem necrosis and severe wilt, 5 = plant death = 100%^59^.

### Molecular and morphological characterizations of the superior isolates

The potent antagonist isolates were characterized by differential morphological traits on ISP2, ISP3, and ISP4 media, melanin formation, growth on medium supplemented with 6 and 10% NaCl, and growth in high temperature (42 °C)^60,61^. DNA extraction was performed according to the method described by Tripathi and Rawal^62^. Polymerase chain reaction (PCR) amplification was performed using the primers 27F: 5′-AGAGTTTGATCCTGGCTCAG-3′ and 1525R: 5′ AAAGGAGGTGATCCAGCC-3′ as described by Chun and Goodfellow^63^. 16S rRNA gene sequences corresponding to IT20 and SS14 were deposited in the GENBANK database under the accession numbers of MK858186 and MH041316 respectively. The sequences were aligned manually with corresponding sequences of available *Streptomyces* species deposited in the GENBANK, EMBL, and DDBJ databases using BLAST search tool.

### Dynamics of the culturable microbiome in the rhizosphere

Fifteen days after pathogen inoculation, the plants were removed carefully and shaken gently. Soil adhering to the roots was considered as the rhizosphere. Rhizosphere samples were collected in sterile zip-lock polyethylene bags. Serial dilutions 1/100 (10^–2^) for counting the most abundant and common soil fungi (e.g. *Penicillium* type colonies), 1/10,000 (10^–4^), and 100,000 (10^–5^) of each sample were prepared after soil suspension in the sterile saline serum (NaCl 0.9%). To obtain the total number of bacterial and fungal colonies respectively, 100 µl of each dilution was spread on the surface of TSB agar and PDA media supplemented with chloramphenicol (250 mg/l) to avoid bacterial contamination.

### Total DNA extraction, amplicon generation, and MiSeq sequencing

A total of 18 samples obtained from six treatments (three replicates per each treatment) were selected for amplicon PCRs and Illumina Next-generation sequencing. Total DNA was extracted from 400 mg soil with PowerSoil DNeasy Isolation Kit (QIAGEN) according to the manufacturer’s manual. DNA integrity was assessed after electrophoresis on 1% agarose gel. Total DNA was quantified by fluorometry using a Quant-iT PicoGreen dsDNA Assay Kit (INVITROGEN, Cergy-Pontoise, France) following the manufacturer’s instructions. Amplicons were generated in two steps according to Berry et al.^64^. The two-step PCR reaction was performed in the final volume of 15 μl containing 7.5 μl PCR Master Mix, 0.25 μM from each initiator, 250 ng T4 gp32 (MPBIO), and 1 ng of DNA. The hypervariable region of bacterial 16S rRNA gene (V3–V4) was amplified by PCR using the fusion primers U341F (5′-CCTACGGGRSGCAGCAG-3′) and 805R (5′-GACTACCAGGGTATCTAAT-3′), with overhang adapters (forward: TCGTCGGCAGCGTCAGATGTGTATAAGAGACAG, adapter: GTCTCGTGGGCTCGGAGATGTGTATAAGAGACAG)^65^. Thermocycler conditions included 98 °C for 3 min and 25 cycles 98 °C for 30 s, 55 °C for 30 s, and 72 °C for 30 s with a final extension of 72 °C for 10 min. PCR products are used as a template for the second step of PCR reaction. In the second step, PCR sequencing was performed using a unique Multiplex primer pair for each sample (barcode). The reaction was performed at 30 μl volume containing 15 μl Phusion High-Fidelity PCR (THERMO FISHER SCIENTIFIC), 1 μl from the forward starter, 1 μl from the reverse Multiplex starter, and 6 μl from the first step PCR product. Thermocycler conditions were 98 °C for 3 min and then the eight-cycle 98 °C for 30 s, 55 °C for 30 s, and 72 °C for 30 s, with the final extension at 72 °C for 10 min. Duplicate PCR product of the second phase was pooled and visualized in agarose gel (2%) to confirm the size of the amplicons (around 630 bp). The amplicons were purified and mixed using the sequalPrepTM Normalization plate kit 96-well kit (INVITROGEN). MiSeq Sequencing (ILLUMINA, 2 × 250 bp) was performed using the MISEQ v2 kit (500 cycles). De-multiplexing and trimming of Illumina adaptors and barcodes were done with ILLUMINA MISEQ REPORTER software (version 2.5.1.3).

### Bioinformatics analysis of 16S rRNA gene diversity

The forward and reverse sequences (R1 and R2) were assembled using PEAR^66^. The quality checks were conducted using the QIIME pipeline^67^ and short sequences were discarded (< 400 bp). Reference-based and de novo chimera detection and clustering (the identity thresholds 94%) of operational taxonomic units (OTUs) were performed using VSEARCH^68^ based on reference databases (GREENGENES). The sequences of each OTU were aligned using PYNAST^69^. Taxonomic assignment was done using UCLUST^70^ and the latest released green genes database (v.05/2013^71^). Sequences were deposited to the SRA at NCBI under the accession number PRJNA665461. The diversity indices used to describe the changes of communities including Phylogenetic Diversity, Shannon, species richness (observed species, Chao), and evenness (Simpson’s reciprocal, equitability) calculated on rarefied OTU table. Weighted UniFrac distance matrices^72,73^ were computed to detect variations in the composition of bacteriome.

### Statistical analysis

Statistical analysis was performed using analysis of variance (ANOVA) by SPSS version 22.0 (SPSS INC. Chicago, IL USA) packages. CFU data were presented on a log scale. The significant difference between treatments was evaluated using Duncan test at the level of *P* < *0.05*. Welch’s t-tests applied to compare abundance data of the top genera significantly differed between two bacterial treatments^74^. Spearman’s rank correlation coefficient was used to evaluate the correlations between selected rhizosphere genera and disease suppression. The redundancy analysis (RDA) was done to evaluate the relationships between treatments, disease suppression, and microbial genera. Permutation multivariate analysis (PERMANOVA) was performed using the Bray–Curtis distance with the function “adonis” within vegan package of R software (version 3.6.1).

## Supplementary Information

Supplementary Figures.
