# Author Correction: *Streptomyces* strains modulate dynamics of soil bacterial communities and their efficacy in disease suppression caused by *Phytophthora capsici*

**DOI:** 10.1038/s41598-021-97719-0

**Published:** 2021-09-07

**Authors:** Sakineh Abbasi, Ayme Spor, Akram Sadeghi, Naser Safaie

**Affiliations:** 1 Department of Plant Pathology, Faculty of Agriculture, Tarbiat Modares University, Tehran, Iran; 2 Department of Agroecology, AgroSup Dijon, INRA, University de Bourgogne, University de Bourgogne Franche-Comte, Dijon, France; 3 Department of Microbial Biotechnology, Agricultural Biotechnology Research Institute of Iran (ABRII), Agricultural Research, Education and Extension Organization (AREEO), Karaj, Iran

Correction to: *Scientific Reports* https://doi.org/10.1038/s41598-021-88495-y, published online 29 April 2021

The original version of this Article contained errors in the spelling of the authors Sakineh Abbasi, Ayme Spor, Akram Sadeghi & Naser Safaie which were incorrectly given as Abbasi Sakineh, Spor Ayme, Sadeghi Akram & Safaie Naser respectively.

The original Article has been corrected.

