# Supplementary material for: Streptomyces strains modulate dynamics of soil bacterial communities and their efficacy in disease suppression caused by Phytophthora capsici
Source: Sci Rep. 2021 Apr 29;11:9317. doi: 10.1038/s41598-021-88495-y (PMC8085009; doi:10.1038/s41598-021-88495-y)
Supplement: Supplementary file 1 — Supplementary Figures. [file 41598_2021_88495_MOESM1_ESM.docx]

***Streptomyces* strains modulate dynamics of soil bacterial communities and their efficacy in disease suppression caused by *Phytophthora capsici***

Sakineh Abbasi^1^, Ayme Spor^2^, Akram Sadeghi^3^*, and Naser Safaie^1^ *

*Correspondent authors

1. Abbasi Sakineh: sa.abbasi@modares.ac.ir/abbasi11368@gmail.com; Department of Plant Pathology, Faculty of Agriculture, Tarbiat Modares University, Tehran, Iran

2. Spor Ayme: Ayme.spor@inrae.fr; Department of Agroecology, AgroSup Dijon, INRA, University de Bourgogne, University de Bourgogne Franche-Comte, Dijon, France

3. Sadeghi Akram: aksadeghi@abrii.ac.ir; Department of Microbial Biotechnology, Agricultural Biotechnology Research Institute of Iran (ABRII), Agricultural Research, Education and Extension Organization (AREEO), Karaj, Iran

1. Safaie Naser: [nsafaie@modares.ac.ir](mailto:nsafaie@modares.ac.ir); Department of Plant Pathology, Faculty of Agriculture, Tarbiat Modares University, Tehran, Iran


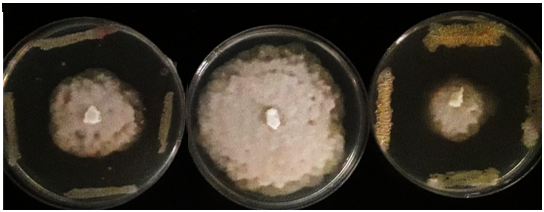


**Figure 1S.** SS14 (left) and IT20 (right) in dual culture assay and growth inhibition effect against *Phytophthora capsici*


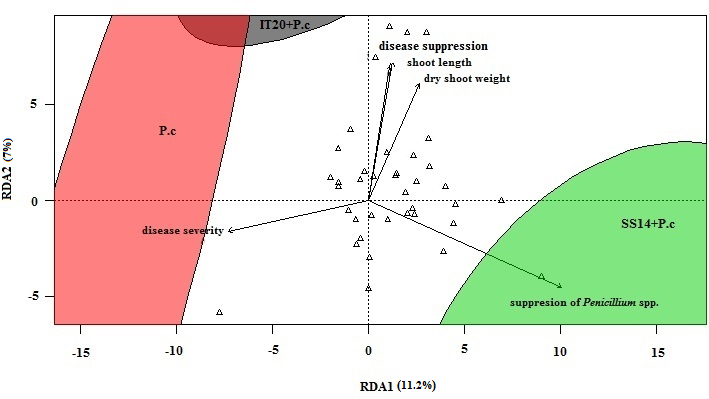


**Figure 2S.** Constrained ordination (redundancy analysis) of bacterial community composition induced by two IT20 and SS14 treatments after inoculation (the most abundant>20%) showing relatedness with environmental factors (direction of vectors shows the correlation of each factor to corresponding treatment). Each point represents the soil community. A significant difference in soil bacterial community composition between two strain treatments was detected (p <0.05, permutation test, 999 permutations)

**Figure 3S**. Total *Penicillium* spp. on PDA media supplemented with lactic acid and chloramphenicol (b) (pH=6.2) in dilutions 10^-2^ after seven days of incubation at 28 ˚C. Values are the means (averaged from three replicates) ±SE. The same letters represent non-significant difference according to Duncan’s Multiple Range Test (*P < 0.05*). C: control; PC: *P. capsici*
